# Comparison of Time-of-Flight and Phase-Shift TLS Intensity Data for the Diagnostics Measurements of Buildings

**DOI:** 10.3390/ma13020353

**Published:** 2020-01-12

**Authors:** Czesław Suchocki

**Affiliations:** Faculty of Civil Engineering Environmental and Geodetic Sciences, Koszalin University of Technology, Śniadeckich 2, 75−453 Koszalin, Poland; czeslaw.suchocki@tu.koszalin.pl

**Keywords:** NDT, TLS, remote defect detection, intensity

## Abstract

In recent years, the terrestrial laser scanning system (TLS) has become one of the most popular remote and nondestructive testing (NDT) methods for diagnostic measurements of buildings and structures as well as for the assessment of architectural heritage. Apart from 3D coordinates, the power of a laser beam backscattered from the scanned object can be captured by TLS. The radiometric information of the point cloud, called “intensity”, can provide information about changes in the physio–chemical properties of the scanned surface. This intensity can be effectively used to detect defects in the surfaces of walls, such as cracks and cavities, moisture, biodeterioration (mosses and lichens) or weathered parts of the wall. Manufacturers of TLS mainly use two different principles for distance measurement, time-of-flight (TOF) and phase-shift (PS). The power of energy in both types of rangefinders might be absorbed or reflected in a slightly different way and provide more or less detailed radiometric point cloud information. The main aim of this investigation is to compare TOF and PS scanners in the context of using TLS intensity data for the diagnostics of buildings and other structures. The potential of TLS intensity data for detecting defects in building walls has been tested on multiple samples by two TOF (Riegl VZ400i, Leica ScanStation C10) and two PS (Z + F 5016 IMAGER, Faro Focus^3D^) scanners.

## 1. Introduction

Structural safety, especially of old and historic buildings, is a very important issue in civil engineering. The geometrical documentation and detection of the visible surface imperfections of buildings and other structures is a key element for their preservation. Many of these buildings and structures often do not have direct access to measurement tools. Therefore, application of well-known and very popular nondestructive testing (NDT) methods, such as rebound hammer test and ultrasonic pulse velocity test are impossible [1,2], because the methods require direct access to the tested element or structure. In this context, data acquisition without any physical contact of the research object is of special interest for measuring the technical condition of architectural structures. With speed and remote acquisition of data, the high accuracy mapping of real objects via a terrestrial laser scanner (TLS) is rapidly becoming one of the most commonly used techniques in the cultural heritage conservation and civil engineering fields as a NDT method. This remote sensing technique, which is based on laser distance measurements, is being widely harnessed as an alternative to photogrammetry technique [3,4]. 

At present, TLS is used in multiple civil engineering applications, such as dam monitoring [5,6], landslide monitoring [7,8], bridge monitoring [9,10], motorway and tunnel monitoring [11,12], façade deformation analysis [13,14], assessing architectural heritage [15,16] and other unusual fields like forestry inventory [17,18], environmental monitoring [19] and crime scene reconstruction [20].

The product of TLS measurements is a three-dimensional (XYZ) point cloud acquired with high-density and high-accuracy. This geometric dataset allows one to build 3D models, as well as detect building defects. However, the TLS can also receive the power of the laser beam backscattered from the observed object, which is called “intensity”. A large number of scientists have focused only on TLS spatial data analysis to detect changes on a building’s surface, such as cracks and cavities [21,22], whereas the intensity data recorded by TLS has been studied by multiple scientists over the last decade [23,24,25,26]. This intensity can be effectively used to identify different changes in the surfaces of walls, such as cracks and cavities [27,28], moisture [29,30] and biodeterioration (mosses and lichens) [31,32]. Currently, the use of the intensity value in diagnostic measurements is of special interest. It should also be noted that, apart from diagnostic measurements intensity data can also be used to identify various elements in point clouds, e.g., in 3D façades modelling [33] or cultural heritage documentations [34,35].

In the author’s opinion, the radiometric information of point clouds should be used in the diagnostic measurement of buildings and structures and should not be separated from geometrical data. 

Manufacturers of terrestrial laser scanners mainly use two different principles for distance measurement between the sensor system and its target. The first principle is time-of-flight (TOF) and second is phase-shift (PS). In the TOF rangefinder, a short laser pulse is emitted towards the target and reflected on its surface; a part of the energy then returns to the scanner detector where the sending and arrival time is measured [36,37,38]. In the PS measurement method, distance is determined by the phase difference between the sent and received waveforms [39,40]. The power of the energy in both types of rangefinders might be absorbed or reflected in a slightly different way. This has a significant effect on the final intensity value, which can be used to detect defects in building walls. 

The goal of this paper was comparison both type of scanners (TOF and PS) in the context of using TLS intensity data in the diagnostics of buildings and structures. The potential of TLS intensity data for the detection and classification of damages in masonry structures of building has been tested on multiple sample. These samples from old historical buildings. Simultaneously, a few practical and technical problems, advantages and disadvantages of both used scanners were noted.

## 2. Motivation 

Recently, the intensity values recorded by TLSs have been increasingly used in multiple fields of civil engineering. So far, authors have conducted thorough and successful research programmes dedicated to harnessing TLS intensity data to detect the saturation of building materials [29,41], using TLS intensity data to detect defects on the building wall [27], or applying TLS geometrical data to detect cracks and cavities in building objects [42]. In these studies, different types of scanners (TOF and PS) from different manufacturers were used. Based on these experiences, was noticed that the collected intensity data from measurements with different scanners are different; in particular, the largest differences occur between TOF and PS scanners. Phase-shift scanners are more sensitive to detecting surface changes such as changes in colour, changes in surface roughness and changes in humidity. The changes in the above-mentioned factors for PS scanners may be more affected by the dispersion and absorption of the laser beam than TOF scanners. Detecting changes in the discontinuities of building surfaces by analysing the intensity data will be easier when the intensity variations are greater. This is extremely important when using algorithms for the automatic selection and classification of a TLS dataset by intensity. Keeping these facts in mind, the present author decided to conduct detailed intensity data tests for the TOF and PS scanner in the diagnostic measurements of buildings. A comparison of the TOF and PS types of scanners (Leica ScanStation C10 and Faro Photon 80) can be found in the work of Alonso et al., (2011) [38], whose study focused on geometric and accurate data analysis. Similar studies regarding the accuracy of the distance measurements by scanners Trimble GX, Leica ScanStation, FARO LS 880 HE, Z+F IMAGER 5006 can be found in the work of Mechelke et al., (2007) [43]. Additionally, in the work [44] one can find comparison of time-of-flight scanners Trimble GS200 from 2003 and Leica Scan Station P20 from 2013. This work focused on the quality data acquisition by both scanners. In all above-mentioned studies were omitted comparisons of TLS intensity data. Moreover, a comparison between TOF and PS scanners in the diagnostic measurements of buildings and structures was not found. Thus, an intensity TLS data test of both types of scanners is required. Apart from a data intensity analysis, the accuracy of the data obtained with the TOF and PS scanners was also compared.

## 3. Theoretical Background of TLS 

In this investigation two TOF (Riegl VZ400i (Horn, Austria)), Leica ScanStation C10, (Sankt Gallen, Switzerland) and two PS (Z+F 5016 IMAGER (Wangen im Allgäu, Germany)), Faro Focus^3D^ (Lake Mary, Florida, USA) scanners were used. The comparisons between the scanners were made in two stages. In the first stage, modern and the most popular in Poland, two TLSs were chosen (Riegl VZ400i and Z + F 5016 IMAGER). In the second stage, one of the most popular in 2010 TLSs were chosen (Leica ScanStation C10 and Faro Focus^3D^). Intentionally selected slightly out-of-date scanners. The scanners from different periods were selected for reliable results.

### 3.1. Radiometric Information of a Laser Beam

TLSs not only provide 3D geometrical information about the measured object but also offer information about the power of the backscatter laser beam by the scanned surface. The portion of energy is recorded by the TLS detector and is called intensity. This intensity, sometimes called reflectance, is represented as the ratio between the emitted and reflected power of the laser wave [45]. The intensity is usually given as a unitless digital number, which depends on the sensitivity of the TLS detector. The TLS detector can usually record intensity with a resolution from 8 to 16 bits (from 2^8^ = 256 to 2^16^ = 65,536). It should also be noted that the TLSs produced by the RIEGL company provide intensity as decibel units. The intensity expressed in decibels (*I_dB_*) can be easily recalculate into a unitless intensity (*I*) as follows [24,46]:

(1) $$I_{dB}=10\log\left(I\right)=10\log\left(\frac{P_{R}}{P_{th}}\right)$$

where *P_R_* is the received power, and *P_th_* is the detection threshold power of the sensor.

The simplified equation for describing the relation between the transmitted signal power (*P_T_*) to the received signal power (*P_R_*) in a TLS is expressed as follows [47]:

(2) $$P_{R}=\frac{\pi P_{T}\rho }{4R^{2}}\eta_{Atm}\eta_{Sys}\cos\left(\Theta \right).$$

Thus, the effect on the intensity of the returning TLS laser beam for the Lambertian surface has a target reflectivity *ρ,* an angle of incidence *Θ*, a range between the TLS and target *R*, an atmospheric transmission factor (*η_Atm_*) and a system transmission factor (*η_Sys_*). The parameters of transmitted signal power and system transmission factor depend on the technical specifications of the TLS and are constant during the measurement. The atmospheric transmission factor can also be accepted as a constant parameter and is negligible during tests [24,48]. Other parameters, such as distance and the incident angle, effect the TLS *intensity* data. Many studies have proven that the effects of changes in these values can be reduced. The literature presents the issues related to reducing the distance and incident angle effects using a mathematic model-driven approach [48,49]. The converted radar range equation is used by above-mentioned method. The second applied approach is based on a data-driven method [24,50]. The data-driven method uses the empirical mathematical model delivered by the raw TLS dataset. The target reflectivity is the last factor affecting the intensity value. The reflectance of a scanned surface depends on the physio–chemical properties of the scanned objects. Thus, Equation (2) can be simplified further [51]:

*Intensity* = *ρ*·*C*_1_·*C*_2_ (3)

where $C_{1}=\pi P_{T}\eta_{Atm}\eta_{Sys}$ is an unknown but constant parameter for a specific scanner and for a specific measurement, and $C_{2}=\frac{\cos\alpha }{4R^{2}}$ is a changeable parameter that can be eliminated.

By properly analysing the intensity value by scanning the building, surface changes, such as roughness, colour and humidity can be detected [25,29,52]. A symptom of the poor technical condition of a building and structure are mainly wall cavities and cracks. It should be noted that a variation of intensity can also be caused by cavities and cracks, because these places are characterized by different roughness and colour in relation to places without defects [27,31]. Apart from recesses of the wall, local discoloration caused by moisture, weathering, salt blooming and biological colonization should also be analysed in the diagnosis of building objects [53,54]. Changes in the roughness and colour of the building’s walls are also caused by these factors. Consequently, they affect the reflection of the laser beam power.

### 3.2. TOF and Phase-Shift Principle Distance Measurement in TLS

One of the most important features of a TLS is that its principles can be used to measure the distance between the TLS sensor and a target. Most TLS applications are mainly determined by the measurement range. There are two types of TLS distance measurements. The first scanning technology is time-of-flight (TOF) and the second is phase-shift (PS). The largest ranges up to a few kilometres can be obtained using the TOF rangefinder of a laser scanner (e.g., *RIEGL* VZ-6000 with a range up to 6 kilometres). Usually, TOF scanners are suitable for long-range measurements, such as topography and mining [55], glacier mapping [56], long range monitoring and archaeology but can also be successfully used for shorter-ranges (e.g., civil engineering) [57]. In contrast, usually TLS measurements based on phase technology can be performed faster (up to 2 million points/sec., for instance Faro FOCUSS 350 PLUS), more accurately, and with a shorter range than TOF scanners. A few years ago, the largest limitation of PS scanners was their range, which did not exceed 100 meters. Currently, the range measurement of phase-shift based technology has grown to above 300 meters. For instance, the Zoller + Fröhlich (Wangen im Allgäu, Germany), Faro (Lake Mary, FL, USA), and Trimble (Sunnyvale, CA, USA) companies specialize in the production of PS scanners (e.g., Z + F IMAGER^®^ 5016, Faro Focus^3D^ 350, Trimble TX8—with measurement ranges up to 365 m, 350 m and 340 m, respectively). Such measuring ranges are sufficient for most different civil engineering applications, such as diagnostic measurements of buildings and structures, as-built measurements, collecting data for building information modelling [58] and cultural heritage conservation [59]. In summary, TOF scanners generally do not have the same performance as PS scanners. The phase difference technique has a medium range, high accuracy, and is ultra-fast, whereas the time-of-flight technique has a longer range but is slightly slower and has slightly less accuracy then the phase difference technique.

The rangefinder based on the TOF distance measurement technique sends out a short laser pulse (e.g., a few ns) towards the target and measures the emitted time and the received time of the laser pulse signal reflected from the target. Thus, the distance (*D*) between the sensor and the target can be described by the formula

(4) $$D=\frac{c}{2}\cdot \Delta t,$$

where *c* is the velocity of light along the path from sensor to target, and Δ*t* is the time interval between the emitted and received laser signal.

The quality of the distance measurement is directly related to the accuracy of the time measurement and the accuracy in detecting the backscattered signal [60]. Thus, atmospheric corrections must be made to improve the measurement precision and accuracy.

An alternative approach to the TOF distance measurement is to use an amplitude modulated a continuous sinusoidal laser beam. In this case, a phase shift distance measurement, the distance that the laser beam travels can be determined by using the phase difference between the reference and return signals [39]. By measuring the phase shift, one can determine the distance (*D*) between the rangefinder and the target using the equation [61,62]

(5) $$D=\frac{c}{2f}\cdot \frac{\varphi }{2\pi },$$

where *c* is the light velocity, *f* is the modulation frequency, and φ is the phase-shift.

## 4. Scope and Methodology

### 4.1. Materials and Methods

**Figure 1:**
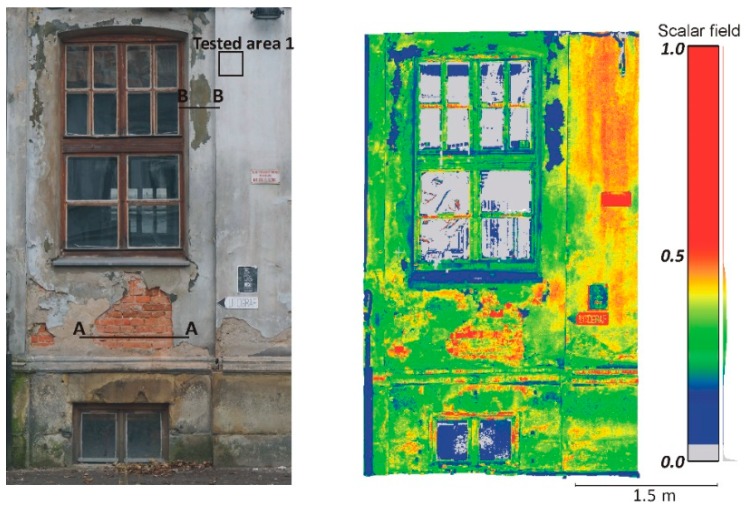
Part of the tobacco factory building—real photo on the left, point cloud on the right.

**Figure 2:**
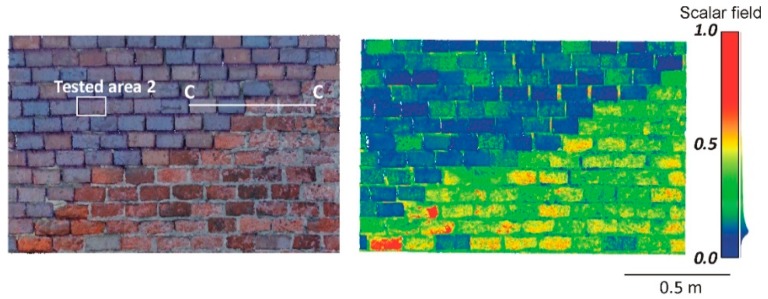
Part of the citadel brick wall—real photo on the left, point cloud on the right.

For a more effective comparison and a better understanding of the differences between the two types of TLSs, two independent measurement campaigns were performed. The first one was conducted out in Cracow in Poland. Two different research objects were used in the study. The first object of research was the old building of a tobacco factory that was part of the Dolne Młyny complex (Cracow, Poland). The façade of the building was in a poor technical state (Figure 1). The second case study was a brick citadel around the Kościuszko Mound (Cracow, Poland). A fragment of the reconstructed brick wall structures was selected for measurement. The selected fragment contained bricks from different periods of time. In this research area, one can find bricks from the 19th and 20th centuries (Figure 2). Both buildings are under the supervision of the conservator. Two different types of TLSs were used during the measurements, a time-of-flight scanner Riegl VZ-400i (Horn, Austria) and a phase-shift scanner Z + F IMAGER 5016 (Wangen im Allgäu, Germany). These scanners are currently the latest generation and up-to-data models. The measurements with both scanners were made one by one from the same measuring station at a distance of approximately 15 m. Moreover, the location of the scanners in relation to the examined object was the same. Thus, the angle of incidence of the laser beam (Θ) and the distance (*R*) between the scanners and the tested object was similar in the two measuring sessions. The measurement with each scanner lasted several minutes, so the atmospheric transmission factor (*η_Atm_*) would be constant for all conducted tests. Therefore, the variable factor affecting the intensity value in both measurements was the technical properties of the TLS, such as the transmitted signal power (*P_T_*) and system transmission factor (*η_Sys_*).

**Figure 3:**
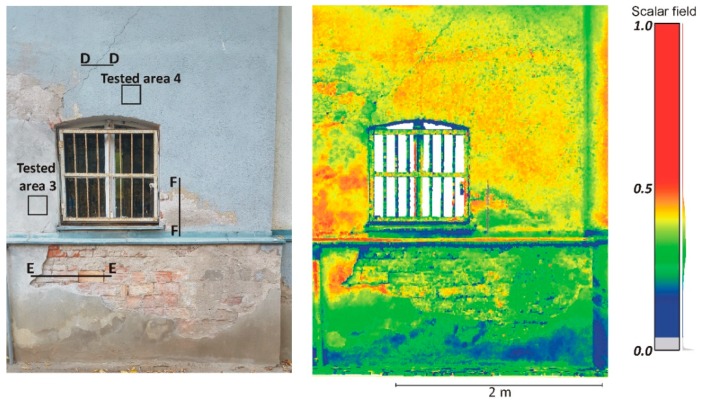
Part of the old historical building—real photo on the left, point cloud on the right.

The second measurement campaign was conducted in Olsztyn (town in Poland). A building with a poor technical condition was used as the research object. This building was a very good sample to survey because it had various defects in its façade, such as cracks, damaged plaster, weathered areas and biological colonization (see Figure 3). Measurements were made in a similar way to the first campaign, but the other two scanners were used. Time-of-flight Leica ScanStation C10 (Sankt Gallen, Switzerland) and phase shift Faro Focus^3D^ scanners (Lake Mary, FL, USA) were used in this survey. These scanners were launched almost one decade ago, so they are slightly out-of-date. 

Technical specifications for the scanners that were tested in the two measurement campaigns are presented in Table 1.

### 4.2. Results of Post-Processing

Firstly, all point clouds from the two measurement campaigns were parsed from their own native formats to *.ptx files. The *.ptx file is a commonly used format for exchanging scan datasets between different software. Thanks to this, the intensity of point clouds from different scanners was fit to the same scale from 0 to 1. Open-source CloudCompare software (version 2.10 - alpha) was used for the post-processing of datasets and to map the results. Point clouds resolution obtained from two different scanners for each sample were slightly different. In the conducted research, the resolution of point clouds of the same sample should be the same. Hence, automatic down-sampling of point clouds by random method were done. In that, the point clouds resolution was the same. Finally, the resolution of point clouds from the first measurement campaign were approximately 4300 points per 0.01 m^2^, and second measurement campaign were approximately 3800 points per 0.01 m^2^.

It should be noted that many researchers recommend point cloud standardization to eliminate the effects of changing the distance and incident angle on the changes of the received signal power in TLS [49,50]. On the other hand, such point cloud standardization is not always necessary. When analysing small areas, this should not cause significant changes in the received signal power. Only small areas are analysed in this investigation, so point clouds standardization are omitted.

#### 4.2.1. Radiometric Analysis of the TLS Point Cloud

**Figure 4:**
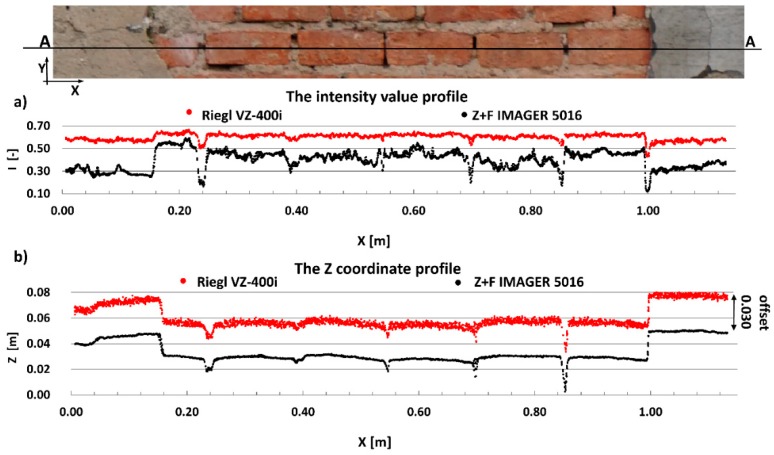
AA profiles—brick wall with damaged plaster: (**a**) intensity data profile and (**b**) geometric data profile.

**Figure 5:**
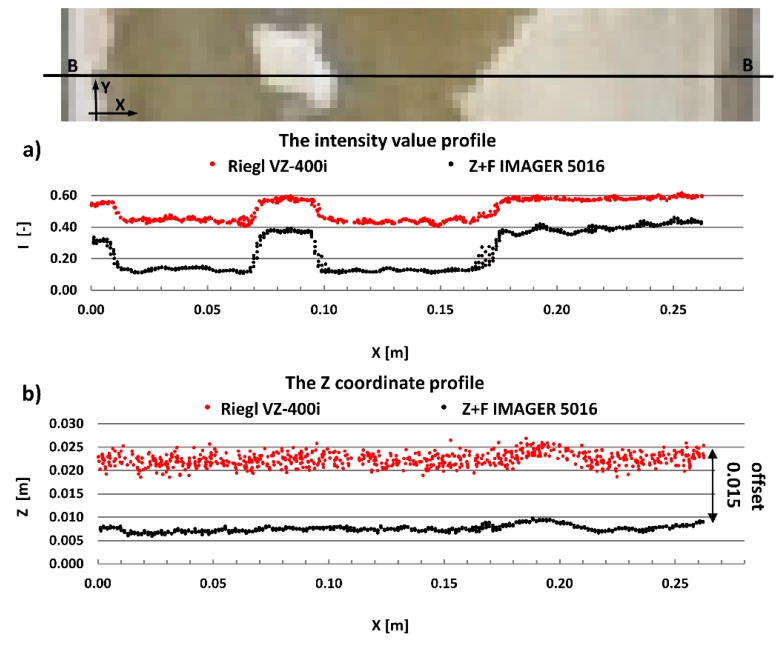
BB profiles—walls with patches of falling paint: (**a**) intensity data profile and (**b**) geometric data profile.

**Figure 6:**
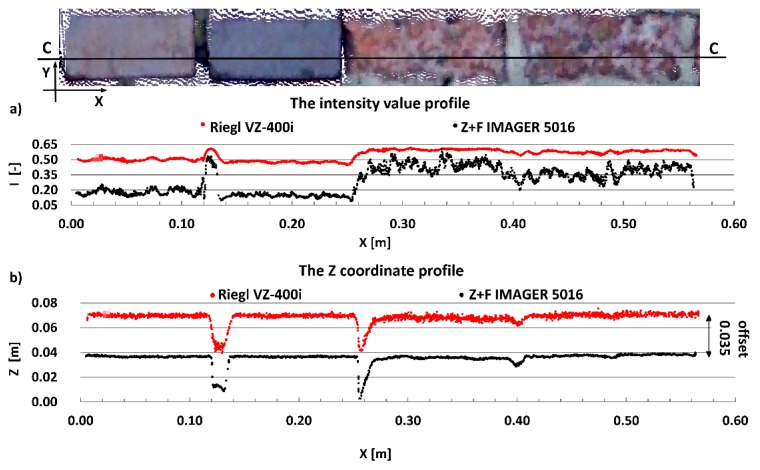
CC profiles—brick walls from different periods of time: (**a**) intensity data profile and (**b**) geometric data profile.

**Figure 7:**
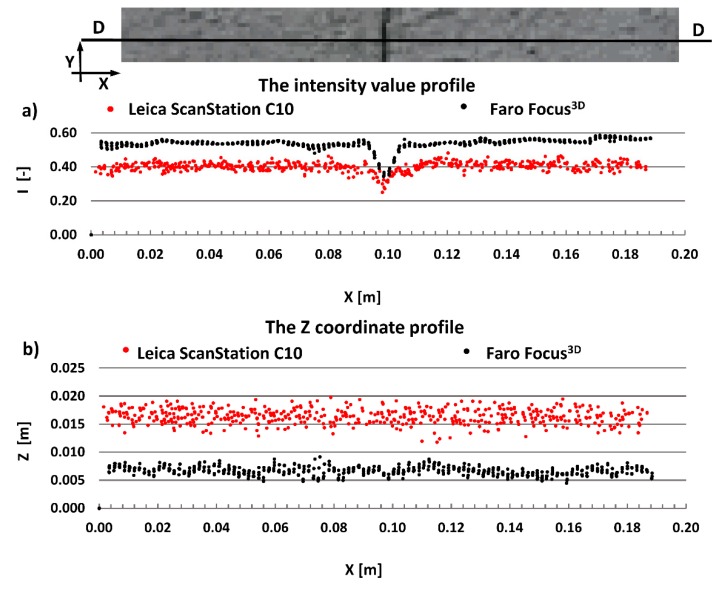
DD profiles—crack of wall: (**a**) intensity data profile and (**b**) geometric data profile.

**Figure 8:**
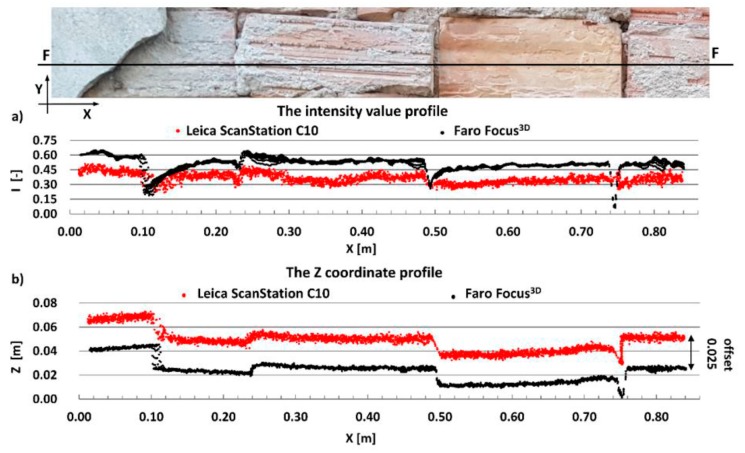
EE profiles—brick wall with damaged plaster, (**a**) intensity data profile, and (**b**) geometric data profile.

**Figure 9:**
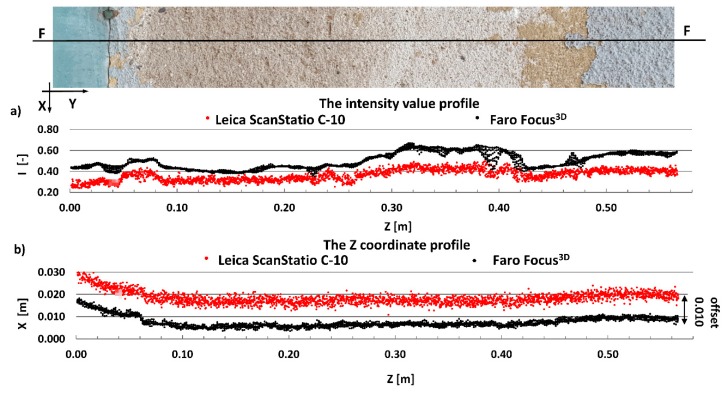
FF profiles—weathered part of the wall: (**a**) intensity data profile and (**b**) geometric data profile.

The main aim of this research is to compare radiometric information provided by different types of TLSs (TOF and PS). Thus, a detailed analysis was undertaken by making profiles from narrow strips 0.01 m wide in the interest area of the research object (Figure 4, Figure 5, Figure 6, Figure 7, Figure 8 and Figure 9). The profiles show an intensity value corresponding to the surface of the wall for both scanners in the same coordinate system OXI (where I = intensity). In addition, the distribution of the points on the examined surface in the OXZ coordinate system is presented in the second profile. In order to clearly present points in second profile for the Z coordinate, an offset was adopted. In total, six profiles were made, three for the first campaign (Figure 4, Figure 5 andFigure 6) and three for the second campaign (Figure 7, Figure 8 and Figure 9). The following profiles concern the following parts/defects of the wall:

- First campaign (see Figure 1 and Figure 2)
  ✓ AA profile—brick wall with damaged plaster
  ✓ BB profile—wall with a patch of falling paint
  ✓ CC profile—brick walls from different periods of time
- Second campaign (see Figure 3)
  ✓ DD profile—crack in the wall
  ✓ EE profile—brick wall with damaged plaster
  ✓ FF profile—weathered part of the wall

The AA profile is an example of the variations in intensity values depending on the wall defects in question. Figure 4a shows that changes in the surface of the wall, from plaster to ceramic brick, affect the variation in intensity. It should also be noted that, by analysing intensity, one can detect the mortar or gaps between the bricks. It is clearly visible that variations of intensity in the area of interest for the Z + F IMAGER 5016 scanner data are approximately two times higher than those for the Riegl VZ-400i scanner data. Thus, changes in the physicochemical properties of the material (i.e., colour and roughness) more strongly affect the variations in the intensity data captured by the PS Z+F IMAGER 5016 scanner than by the TOF Riegl VZ-400i scanner. By analysing geometrical data (Figure 4b), it can be concluded that the data captured by the Riegl VZ-400i scanner have more noise than the data captured by the Z + F IMAGER 5016 scanner. The main reason is the different ranging error in both scanners (see Table 1). The laser spot size may also effect on the noise.

The next example concerns changes in the wall surfaces caused by a lack of paint. A lack of paint on the wall significantly affects the reflectivity properties of the material, which is clearly visible in Figure 5a. In an area where there is no paint, the intensity value decreases significantly. Similar to the previous example, changing the surface of the wall has a greater effect on the intensity of the point cloud captured by the Z + F IMAGER 5016 scanner than by the Riegl VZ-400i scanner. Figure 5b shows the spatial distribution of the points where each profile was created. One can see how much noise the Riegl VZ-400i scanner has. It can be concluded that the Riegl VZ-400i scanner has a greater error range then the Z + F IMAGER 5016 scanner.

A fragment of the reconstructed ceramic brick wall was the last example used in the first campaign. Figure 6 presents the CC profile. The two bricks on the left are from the 20th century, and the two on the right are from the 19th century. These bricks differ significantly in their colour and roughness. The bricks from the 19th century are brighter and more rough. These factors had an effect on their intensity value, as shown in Figure 6a. The intensity obtained with the Z + F IMAGER 5016 scanner on bricks from the 19th century, however, suffers from considerable noise due to the discoloration and variable roughness. The test results of this example also confirm the greater sensitivity of the Z + F IMAGER 5016 scanner to changes in the physicochemical properties of the scanned surface than the Riegl VZ-400i scanner.

The first analysed example in the second campaign was a crack on the building wall. The profile was made perpendicular to the crack. As shown in Figure 7b, the geometric data captured by both scanners do not provide information about the crack. The width of the crack was too small in relation to the laser spot size (see Table 1), hence this crack was impossible to register by scanners. On the other hand, the crack was sufficient to absorb the laser beam. This phenomenon is clearly visible in Figure 7a, where a decreased intensity value occurred at the crack site. Thus, a geometric analysis of the point cloud does not allow the detection of narrow cracks, whereas a radiometric analysis of the point cloud does allow the detection of such cracks. By comparing the results of the intensity between both scanners (Figure 7a), one can see that the Faro Focus^3D^ scanner more clearly highlighted the crack than the Leica ScanStation C10 scanner. 

The next example concerns the ceramic brick wall with damaged plaster. In Figure 8a, there is a clearly visible difference between the values of the intensity achieved for plaster and brick by the Faro Focus^3D^ scanner. This difference is much less visible for the Leica ScanStation C10 scanner. The surface changes and fugues between bricks are very well highlighted by the Faro Focus^3D^ scanner. Analysing the spatial distribution of the points in Figure 8b, it can be concluded that the Faro Focus^3D^ scanner is more accurate and has less noise.

The last example shows the weathered part of the wall. Similar to the previous example, the intensity dataset captured by the Faro Focus^3D^ scanner better highlights changes in the surface than the intensity dataset captured by the Leica ScanStation C10 scanner (see Figure 9a). As shown in Figure 9b, the geometric data captured by the Leica ScanStation C10 scanners have more noise than the data captured by the Faro Focus^3D^ scanners. The main reason is that the ranging error for the Faro Focus^3D^ scanner is twice smaller than for the Leica ScanStation C10 scanner (see Table 1). 

The intensity values for individual samples captured by the tested scanners are presented in Table 2. Based on the minimum and maximum intensity, the range for the two measurement sessions was calculated and compared. The range of intensity value for the PS scanners is always greater than that for TOF scanners (see column 3 in Table 2). For instance, the biggest difference in the intensity range for the first campaign between the Riegl VZ 400i scanner and the Z + F Imager 5016 scanner occurred in the CC profile (a difference of 3.04 times). A similar relationship is visible in the second campaign. The biggest difference in the intensity range between the Leica ScanStation C10 scanner and the Faro Focus^3D^ scanner occurred in the DD profile (2.02 times). Hence, the laser beams in PS scanners are more sensitive to physicochemical changes in the scanned surface of the building wall than in the TOF scanners. The registered value of *intensity* is mainly affected by use of two different type of rangefinder, power of the emitter and sensitivity of the detector. Wavelength of laser beam may also affect the intensity value.

#### 4.2.2. Geometric Analysis of the TLS Point Cloud

**Figure 10:**
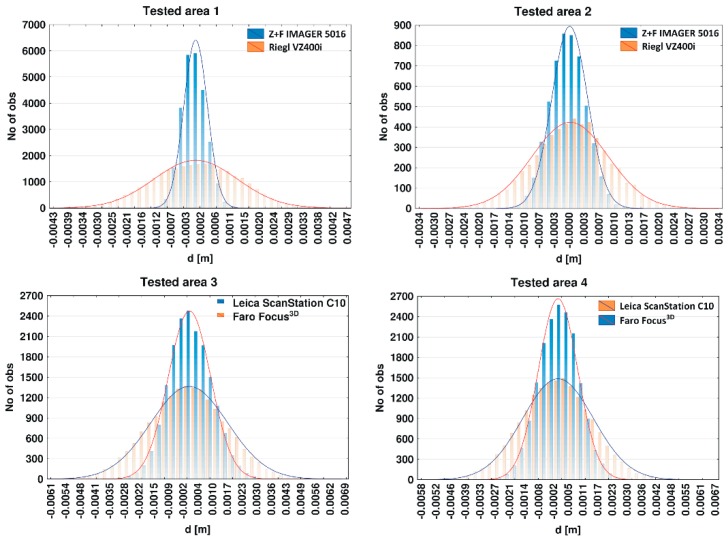
Histograms of distance measurement errors of the tested areas.

Assessment of the geometrical data provided by the PS and TOF scanners was conducted in the four selected test areas (see Figure 1, Figure 2 and Figure 3). The selected areas were characterized by a flat and homogeneous surface containing no anomalies. The reference planes were fitted based on the point cloud coordinates of each tested area. To determine the optimum reference plane (*π*) of the analysed area, an algorithm based on the Mean Sum Error (MSE) presented by Chen [63] was used. Then, the distances *d_i_* of all points from reference plane *π* are calculated. The *d* value was adopted as an error of the distance measurement of scanners and submitted for statistical analysis. Table 3 provides the results of the statistical analyses. In addition, the *d* value distribution of each tested area for the PS and TOF scanners is presented in histograms (Figure 10).

Table 3 contains the minimum and maximum values, range, and standard deviation. Based on the results in Table 3, the range (column 4) for the Riegl VZ400i scanner is always significantly larger than that for the Z + F IMAGER 5016 scanner in tested areas 1 and 2. For tested areas 1 and 2, the range is two-times and three-times larger, respectively. Greater range means that the scanner performed a less accurate distance measurement. A similar relationship occurs in the standard deviation (column 5), which can be taken as a plane fitting error. For instance, the standard deviation for the Z + F IMAGER 5016 and Riegl VZ400i in tested area 1 is equal to 0.0004 m and 0.0013 m, respectively, and tested area 2 is equal to 0.0004 m and 0.008 m, respectively. Histograms of the distance measurement error of the tested areas also confirm a more favourable distribution for the Z + F IMAGER 5016 scanner than for the Riegl VZ400i scanner.

A similar situation exists between the Faro Focus^3D^ scanner and the Leica ScanStation C10 scanner. In this case, the differences between the TOF and PS scanners are slightly smaller. For instance, the range (column 4) for the Faro Focus^3D^ scanner and ScanStation C10 scanner are equal 0.0074 m and 0.0126 m in tested area 3 and equal to 0.0061 and 0.0125 in tested area 4. Conversely, the standard deviation in tested area 3 and 4 is equal, totalling 0.0008 m for the Faro Focus^3D^ scanner and 0.0015 m for the Leica ScanStation C10 scanner.

In summary, in both measurement campaigns, PS scanners are characterized by a much greater precision of the distance measurement in relation to the TOF scanners. Ranging errors for tested PS scanners are Z + F IMAGER 5016: ±1 mm + 10 ppm/m and Faro Focus^3D^: ±2 mm, for tested TOF scanners are Riegl VZ-400i: ±5 mm and Leica ScanStation C10: ±4 mm. The obtained test results were consistent with the ranging error of the scanners provided by the manufacturer.

## 5. Discussion

The greater sensitivity of the PS scanner laser detector in relation to the TOF scanner laser detector will facilitate easier detection of building defects. This should influence more efficient segmentation and classification of the point cloud for the diagnostic measurements of buildings and structures. 

**Figure 11:**
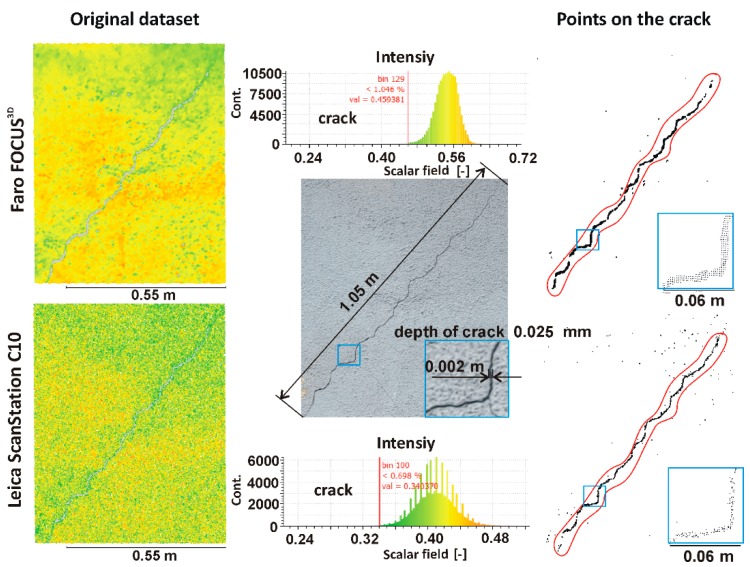
2D crack mapping by segmentation.

Building cracks are a common symptom of technical deterioration. Thus, crack detection is a very important issue in the diagnostic measurements of buildings and structures. Keeping this fact in mind, the present author decided to analyse the wall crack by segmenting the dataset. Based on the intensity value analysis, manual classification was performed. In the place where the crack occurs, the intensity value is significantly decreased. Hence, in the research area, a low intensity value dataset was separated. Thus, the separated measuring points were on the crack. Figure 11 and Table 4 present the results of the segmentation of the point clouds obtained by the Faro Focus^3D^ scanner and Leica ScanStation C10 scanner.

Comparing the segmentation results by intensity, one can see that more information about crack was provided by the point cloud captured by Faro Focus^3D^. Leica ScanStation C10 shows 1025 points on the crack, whereas Faro Focus^3D^ shows 1655 points on the crack. It should be noted that both input datasets had the same number of points. More points on the crack allows for better dimension validation of the crack. Thus, in this analysis, the phase-shift Faro Focus^3D^ scanner was the better device in building diagnostics. Surface crack identification and crack width and depth determination have play a very important roles for properly technical diagnostic of buildings and structures. Defects detection and determination of their size mainly depends on the location of the TLS in the relation to the wall defects, and technical parameters of TLS such as laser spot size. These relationships were well presented in the paper [21,64]. It should also be noted that very good solution assessment of cracks on building walls is done using laser scanning survey supported by digital image processing [65].

**Figure 12:**
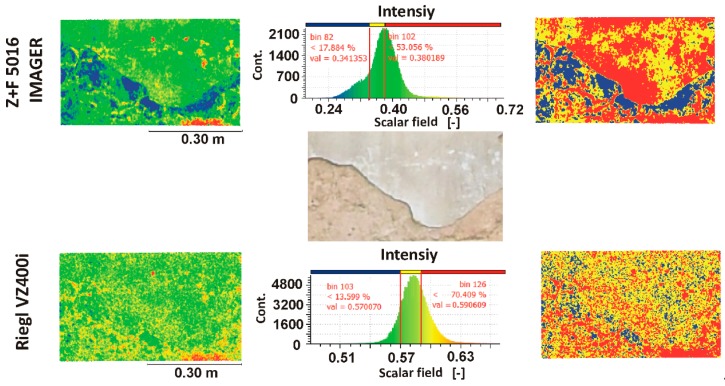
2D damaged plaster mapping by point cloud classification.

The plaster defect was the second example used in data classification. The purpose of this problem was to detect a border between the undamaged and damaged part of the plaster. For this purpose, point clouds from the measurements of the Riegl VZ 400i scanner and Z+F Imager 5016 scanner were used. In the tested area, the intensity data was divided into three groups (see histograms in Figure 12). As a result of this process, the searched border should be more visible. The change the intensity value in this case is mainly caused by a change in roughness of surface wall. Based on the manual classification of the intensity values, changes in the surface of the tested wall can be detected.

According to the results provided by the manual classification and visual inspection, one can see that the border between the damaged and undamaged plaster is clearly highlighted based on the intensity data captured by the Z+F Imager 5016 scanner. In the case of the Riegl VZ 400i scanner, this could not be achieved. Thus, the sensitivity of the detector of the Riegl VZ 400i scanner was too low. Consequently, in this analysis, the phase-shift Z+F Imager 5016 scanner was the clear winner. 

In both presented cases of point cloud classification, the intensity dataset obtained by phase-shift scanners provided more useful information on building defects than time-of-flight scanners. Thus, phase-shift scanners are more suitable for the diagnostic testing of buildings and structures.

A more sensitive detector provides more useful information to detecting wall surface changes. Many studies have shown that radiometric information of the point cloud can be effectively used to damage detection of buildings and structures [31,66].

## 6. Conclusions

Through a selected study, this paper demonstrates a test of the PS and TOF terrestrial laser scanners on real objects and compares the intensity data and geometric accuracy of the information obtained by the Riegl VZ 400i, Z + F Imager 5016, Faro Focus^3D^, and Leica ScanStation C10. By means of the radiometric information provided by PS scanners (Z+F Imager 5016 and Faro Focus^3D^), damage to the façades was accurately mapped significantly better than using the radiometric information provided by TOF scanners (Riegl VZ 400i and ScanStation C10). A similar relationship has been noted in the precision of distance measurement, but these results are a consequence of the technical specifications of the tested scanner. It is well known that PS scanners measure distances more accurately than TOF scanners. In this investigation, detailed analyses of radiometric information provided by TOF and PS scanners were conducted. The test results clearly show that used PS scanners provide much more information about the physicochemical properties of the tested surface compared to the used TOF scanners. In general, it can be assumed that PS scanners are slightly better instruments for detecting the imperfections in building walls. This is due to their higher precision, and more detailed radiometric data. Nevertheless, TOF scanners cannot be excluded from these types of building diagnostic measurements.

## Figures and Tables

**Table 1:** Comparison of the selected scanner parameters.

	**The First Measurement Campaign**		**The Second Measurement Campaign**	
	Riegl VZ-400i 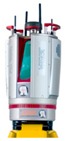	Z + F IMAGER 5016 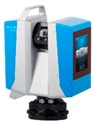	Leica ScanStation C-10 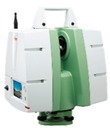	Faro Focus^3D^ 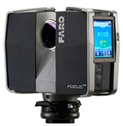
Year of Production	2018	2018	2010	2010
Type of Rangefinder	time-of-flight	phase-shift	time-of-flight	phase-shift
Wavelength	near infrared	-	532 nm	905 nm
Scan Rate Points/sec	500,000 (1200 kHz)	1,100,000	Up to 50,000	1,000,000
Range	800 m @ 90%	365 m	300 m @ 90%	120
Beam Diameter	-	3.5 mm at exit	From 0 to 50 m: 4.5 mm (FWHH-based), 7 mm (Gaussian-based)	3 mm at exit
Ranging Error	5 mm	±1 mm + 10 ppm/m	±4 mm within a 1–50 m range	± 2 mm
Beam Divergence	0.35 mrad	0.3 mrad	–	0.19mrad

**Table 2:** Summary of the basic statistics of the intensity values for the tested scanners.

Intensity	Firs Campaign			Second Campaign		
	Riegl VZ 400i [1]	Z+F Imager 5016 [2]	[2]/[1] [3]	Leica ScanStation C10 [1]	Faro Focus^3D^ [2]	[2]/[1] [3]
	AA Profile			DD Profile		
Minimum	0.42800	0.11960		0.21948	0.06274	
Maximum	0.66571	0.59395		0.51001	0.64868	
Range	0.23771	0.47436	2.00	0.29053	0.58594	2.02
	BB Profile			EE Profile		
Minimum	0.40514	0.10644		0.25000	0.25399	
Maximum	0.61600	0.46113		0.48242	0.58618	
Range	0.21086	0.35469	1.68	0.23242	0.33219	1.43
	CC Profile			FF Profile		
Minimum	0.44457	0.09145		0.24048	0.35039	
Maximum	0.61486	0.60849		0.49536	0.66914	
Range	0.17029	0.51704	3.04	0.25488	0.31875	1.25

**Table 3:** Errors of the distance measurement of the time-of-flight (TOF) and phase-shift (PS) scanners basic statistics.

	No of obs. [1]	Minimum [2]	Maximum [3]	Range [4]	Std.Dev. [5]
	***Tested Area 1***				
Z+F IMAGER 5016-PS	25787	−0.0013	0.0014	0.0027	0.0004
Riegl VZ400i-TOF	25787	−0.0044	0.0047	0.0090	0.0013
	***Tested Area 2***				
Z+F IMAGER 5016-PS	5350	−0.0013	0.0015	0.0028	0.0004
Riegl VZ400i-TOF	5350	−0.0032	0.0033	0.0064	0.0008
	***Tested Area 3***				
Faro Focus^3D^-PS	17857	−0.0036	0.0038	0.0074	0.0008
Leica ScanStation C10-TOF	17857	−0.0058	0.0067	0.0126	0.0015
	***Tested Area 4***				
Faro Focus^3D^-PS	17857	−0.0030	0.0031	0.0061	0.0008
Leica ScanStation C10-TOF	17857	−0.0058	0.0067	0.0125	0.0015

**Table 4:** Comparison of the point numbers of the original and segmented point clouds.

	Original Dataset				Points on the Crack			
	No of points	Intensity min.	Intensity max.	Range	No of points	Intensity min.	Intensity max.	Range
Faro Focus^3D^	172917	0.18774	0.72681	0.53906	1655	0.18774	0.46000	0.27226
Leica ScanStation C10	172917	0.22192	0.52515	0.30322	1025	0.22192	0.34000	0.11808
